# CRISPR1 analysis of naturalized surface water and fecal *Escherichia coli* suggests common origin

**DOI:** 10.1002/mbo3.348

**Published:** 2016-03-22

**Authors:** Lisa D. Tymensen

**Affiliations:** ^1^ Irrigation and Farm Water DivisionAlberta Agriculture and ForestryAlbertaCanada

**Keywords:** CRISPR1, *Escherichia coli*, ET‐1 clade, MLST, naturalized, surface water

## Abstract

Clustered regularly interspaced short palindromic repeats (CRISPRs) are part of an acquired bacterial immune system that functions as a barrier to exogenous genetic elements. Since naturalized *Escherichia coli* are likely to encounter different genetic elements in aquatic environments compared to enteric strains, we hypothesized that such differences would be reflected within the hypervariable CRISPR alleles of these two populations. Comparison of CRISPR1 alleles from naturalized and fecal phylogroup B1 *E. coli* strains revealed that the alleles could be categorized into four major distinct groups (designated G6–G9), and all four allele groups were found among naturalized strains and fecal strains. The distribution of CRIPSR G6 and G8 alleles was similar among strains of both ecotypes, while naturalized strains tended to have CRISPR G7 alleles rather than G9 alleles. Since CRISPR G7 alleles were not specific to naturalized strains, they, however, would not be useful as a marker for identifying naturalized strains. Notably, CRISPR alleles from naturalized and fecal strains also had similar spacer repertoires. This indicates a shared history of encounter with mobile genetic elements and suggests that the two populations were derived from common ancestors.

## Introduction

Although the existence of naturalized *E. coli* that persist and multiply in aquatic environments is supported by an increasing number of studies (Ishii and Sadowsky 2008), the fundamental question remains of whether these naturalized *E. coli* represent an autochthonous population (i.e., self‐sustaining in the absence of fecal input) or whether they are environmentally selected fecal contaminants. Although some strains associated primarily with aquatic environments have shown to be phylogenetically divergent from *E. coli*, such as those from cryptic clades III‐V (Walk et al. 2009; Clermont et al. 2011), most studies suggest that naturalized aquatic populations are predominated by phylogroup B1 strains (Ratajczak et al. 2010; Berthe et al. 2013; Tymensen et al. 2015). However, phylogroup B1 strains are also abundant in the feces of certain livestock and wildlife (Higgins et al. 2007), making it difficult to determine whether naturalized *E. coli* populations are truly autochthonous. To address this, we compared the genetic relatedness of CRISPR (clustered regularly interspaced short palindromic repeats) arrays of naturalized and fecal phylogroup B1 *E. coli*.

CRISPRs canonically serve as part of an adaptive bacterial immune system against foreign nucleic acids (Horvath and Barrangou 2010). They consist of partially palindromic direct DNA repeats separated by spacers that are often derived from foreign genetic elements. The spacers can serve as templates for RNA‐mediated interference with the exogenous genetic elements, thereby limiting horizontal gene transfer. Bacteria continually acquire CRISPR spacers from attacking foreign mobile genetic elements (Yosef and Qimron 2015), ultimately generating a hypervariable spacer repertoire among different strains. This variability has been leveraged for use in genetic typing (Delannoy et al. 2012). Although CRISPR immunity does not appear to be highly active in present‐day *E. coli* (Touchon et al. 2011), their alleles ostensibly reflect historical encounters with exogenous genetic elements. Since naturalized *E. coli* populations have likely encountered different genetic elements in aquatic environments compared to enteric strains residing in the intestine, we hypothesized that these two populations would have different CRISPR spacer repertoires owing to the incorporation of different foreign genetic elements present in their respective environments.

## Experimental Procedures

### Strains

*E. coli* strains were obtained from a previously established collection of surface water, sediment, and fecal strains, that were isolated from the Milk River watershed in Alberta, Canada (Tymensen et al. 2015) (Table 1). The naturalized strains included those from the ET–1 clade, along with other phylogroup B1 strains, and represented several clonal genotypes (based on accessory gene profiles) that were either specific to or numerically more abundant (i.e., overrepresented) in surface water and sediment compared to feces. Fecal strains were largely from cattle, as they were the predominant contributor of fecal contamination in the watershed; however, several ET–1 clade strains from other livestock and wildlife were also included, since few cattle ET–1 clade strains were present in the isolate collection.

**Table 1:** Isolation source, CRISPR allele spacer number and relatedness group, sequence type (ST), and multilocus sequence typing (MLST) data for phylogroup B1 strains analyzed in this study

Strain ID	Source	Spacers/allele	CRISPR relatedness group^a^	ST (MLST)	MLST allele numbers^b^						
					*aspC*	*clpX*	*fadD*	*icdA*	*lysP*	*mdh*	*uidA*
ARDMR005	Surface water	13	G6	1100	7	5	77	4	1	2	261
ARDMR007	Surface water	9	G7	223	5	6	71	2	1	8	1
ARDMR017	Sediment	9	G7	230	5	6	71	25	1	8	1
ARDMR021	Cow	11	G9	619	4	5	2	38	1	5	1
ARDMR022	Cow	18	G9	1171	7	5	5	2	1	8	3
ARDMR023	Cow	0^c^	n/a	311	4	5	2	69	1	72	1
ARDMR024	Cow	8	G6	145	5	5	2	2	1	13	23
ARDMR025	Cow	15	G8	603	32	5	5	133	1	13	23
ARDMR026	Cow	11	G9	619	4	5	2	38	1	5	1
ARDMR037	Surface water	13	G7	223	5	6	71	2	1	8	1
ARDMR041	Surface water	7	UG	1153	4	6	5	2	1	2	1
ARDMR043	Surface water	17	G9	nd	5	5	2	30	1	1	1
ARDMR056	Surface water	8	G9	nd	5	5	2	30	1	12	1
ARDMR058	Surface water	10	UG	nd	32	5	2	38	1	5	1
ARDMR063	Sediment	7	G8	286	4	5	13	87	1	8	5
ARDMR067	Cliff Swallow	16	G9	nd	5	5	2	4	1	5	63
ARDMR068	Cow	10	G6	1114	5	191	2	2	1	12	23
ARDMR070	Cow	6	G6	nd	7	5	13	2	1	20	1
ARDMR075	Cow	19	G8	150	59	5	64	4	1	5	1
ARDMR076	Cow	14	G9	1180	5	5	2	221	1	5	277
ARDMR077	Cow	17	G8	nd	59	5	64	4	1	5	1
ARDMR078	Cow	9	G7	721	5	6	167	25	1	8	1
ARDMR079	Cow	16	G9	nd	5	6	2	4	1	13	63
ARDMR080	Cow	19	G9	148	4	5	2	4	1	5	1
ARDMR081	Cow	14	G7	230	5	6	71	25	1	8	1
ARDMR082	Cow	13	G7	230	5	6	71	25	1	8	1
ARDMR083	Cow	14	G9	1049	5	6	13	4	1	8	257
ARDMR085	Cow	6	UG	630	5	5	2	2	1	8	3
ARDMR086	Cow	13	G9	131	4	6	2	39	1	5	41
ARDMR087	Surface water	13	G6	nd	4	4	2	2	1	12	3
ARDMR088	Surface water	19	G7	230	5	6	71	25	1	8	1
ARDMR090	Surface water	9	G7	1181	5	6	71	222	1	8	1
ARDMR091	Surface water	11	G8	603	32	5	5	133	1	13	23
ARDMR093	Surface water	12	G6	146	5	5	2	2	1	5	23
ARDMR094	Surface water	9	G7	230	5	6	71	25	1	8	1
ARDMR095	Surface water	9	G8	1182	5	5	188	4	134	2	1
ARDMR096	Surface water	13	G7	223	5	6	71	2	1	8	1
ARDMR097	Surface water	8	G9	1183	67	8	2	2	1	5	278
ARDMR098	Surface water	9	G7	230	5	6	71	25	1	8	1
ARDMR099	Surface water	13	G7	223	5	6	71	2	1	8	1
ARDMR100	Surface water	6	G7	230	5	6	71	25	1	8	1
ARDMR101	Surface water	9	G7	1184	182	6	71	25	1	8	1
ARDMR102	Surface water	9	G7	230	5	6	71	25	1	8	1
ARDMR103	Surface water	13	G9	1185	5	5	246	2	1	13	1
ARDMR104	Surface water	16	G6	nd	7	5	5	4	1	5	95
ARDMR105	Surface water	11	G6	145	5	5	2	2	1	13	23
ARDMR106	Surface water	9	G7	230	5	6	71	25	1	8	1
ARDMR107	Surface water	18	G9	1186	4	198	5	4	1	13	23
ARDMR108	Cliff Swallow	9	G7	230	5	6	71	25	1	8	1
ARDMR109	Cow	13	G6	650	7	8	13	2	1	5	23
ARDMR110	Cow	9	G7	721	5	6	167	25	1	8	1
ARDMR111	Cow	8	UG	157	7	5	20	23	1	29	1
ARDMR114	Horse	16	G9	1027	5	110	2	4	1	13	63
ARDMR115	Horse	5	G8	nd	4	6	1	4	1	169	1
ARDMR116	Sheep	23	G9	93	7	6	13	2	1	5	5
ARDMR117	Deer	9	G7	230	5	6	71	25	1	8	1
ARDMR118	Surface water	8	G8	129	7	6	13	4	1	13	23

nd, not determined; ST, sequence type.

a As previously defined by Touchon et al. 2011.

b Allele numbers according to Shigatox EcMLST database.

c CRISPR1 was not amplified from strain ARDMR023.

### CRISPR sequencing and analysis

CRISPR1 arrays of each *E. coli* strain were amplified by PCR using primers flanking the *iap* (C1Fw, GTTATGCGGATAATGCTACC) and *cas2* (C1Rev, CGTAYYCCGGTRGATTTGGA) genes, as previously described (Touchon et al. 2011). Forward and reverse sequencing of the PCR products was conducted by Functional Biosciences (Madison, WI). Consensus sequences were assembled using the Staden package v3.3. (Hinxton, UK) (Staden et al. 2003). Sequences were submitted to GenBank with accession numbers KT821503 to KT821545.

Analysis of CRISPR sequences was performed using the CRISPRdb database and CRISPRtionary tool (Grissa et al. 2007). Spacers were automatically numbered, with each distinct spacer being assigned a different number. A two‐base mismatch for spacers was allowed for distinct spacer assignment. CRISPR1 sequences of representative reference strains from the E. coli reference (ECOR) collection and each of the four CRISPR sequence groups, G6 to G9 (as previously identified by Touchon et al. 2011), were obtained from GenBank.

### MLST

Phylogenetic reconstruction was based on the seven‐gene multilocus sequence typing (MLST) protocol described elsewhere (Shigatox.net). Forward and reverse sequences for each of the seven genes (*aspC*,* clpX*,* fadD*,* icdA*,* lysP*,* mdh*, and *uidA*) were assembled using the Staden package v3.3 (Hinxton, UK). (Staden et al. 2003). New alleles and sequence types were submitted to the Shigatox EcMLST database (Qi et al. 2004). For each *E. coli* strain, consensus sequences for each of the seven genes were concatenated and aligned using MUSCLE (default parameters) (Edgar 2004) as implemented from within Mega5 (Tamura et al. 2011). The alignment was imported into SplitsTree4 v.4.13.1 (Tubingen, Germany) and analyzed according to the Neighbour‐Net algorithm (default parameters) (Huson and Bryant 2006). MLST data of representative *E. coli* strains from the ECOR collection, clade ET1, and several additional non‐phylogroup B1 strains from the Milk River were included in the analysis for comparison purposes (Table S1). MLST sequences for reference strains from the ECOR collection were obtained from the Shigatox EcMLST database. Dr. Seth Walk (Montana State University, Bozeman, MT) kindly provided the MLST sequences for the reference clade ET–1 strains.

## Results and Discussion

**Figure 1:**
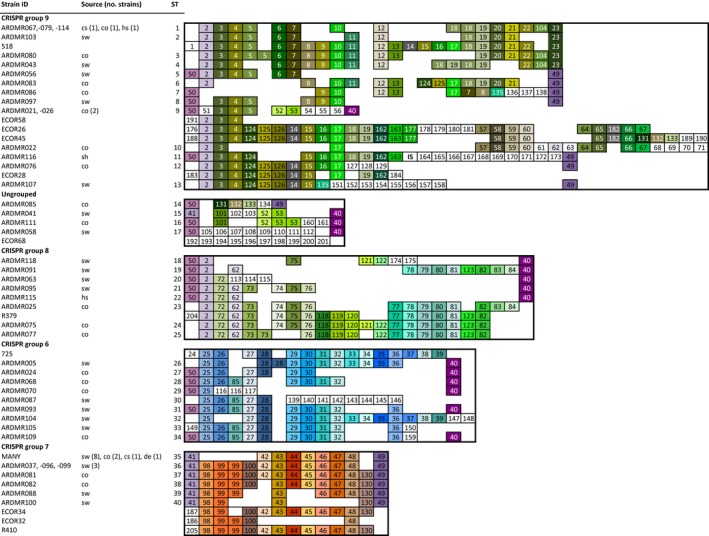
Graphical representation of CRISPR1 alleles from fecal and naturalized *E. coli*. Each spacer is represented by a square (direct repeats not shown). Identical spacers found in two or more strains have identical numbers and colors, while strain‐specific spacers are white. Alleles were aligned with the most ancient spacers on the left. Gaps were introduced to improve spacer alignment. Isolate source and CRISPR sequence types (ST) are shown. Spacers were grouped according to four different spacer repertoire relatedness groups (G6 to G9, as previously defined by Touchon et al. 2011). ‘MANY’ includes strains ARDMR007, −017, −078, −090, −094, −098, −101, −102, −106, −108, −110, and −117. Reference strains were from the ECOR collection or Touchon et al. 2011 (strains 518, R379, 725, and R410). cs, cliff swallow; co, cow; hs, horse; sw, surface water/sediment; sh, sheep; de, deer; IS, insertion sequence.

CRISPR1 arrays of 56 fecal and naturalized *E. coli* strains from a previous study (Tymensen et al. 2015) were sequenced (see Table 1). CRISPR sequence analysis identified a total of 177 distinct spacers among the surface water and fecal *E. coli* strains (Fig. 1). Among naturalized strains, 68 of the 74 common spacers, which were defined as being present in two or more strains, were also present in fecal strains, indicating that common spacer repertoires were largely similar (Table S2), which was contrary to our hypothesis. The spacers were arranged as 40 different alleles, with only one allele (ST35) that was shared by naturalized and fecal strains (Fig. 1). Allelic variation among naturalized strains was largely due to spacer deletion, where alleles from naturalized strains were similar to those of fecal strains, but missing spacers. The remaining variation could be attributed to the presence of strain‐specific spacers in eight of the 21 alleles.

**Figure 2:**
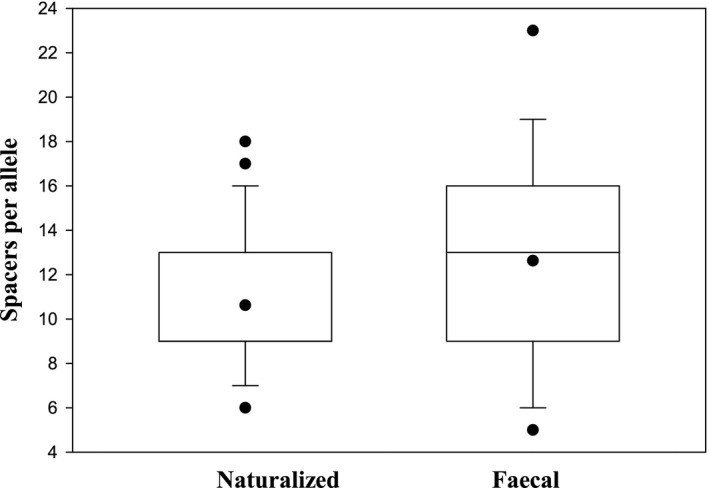
Box plot of the number of spacers in CRISPR alleles of naturalized versus fecal *E. coli*. Dot within the box represents mean. Mean ± SD for naturalized: =11 ± 3 spacers/allele versus fecal: =13 ± 5; Mann–Whitney Rank Sum Test: *P *=* *0.08, considered not significant.

CRISPR alleles from naturalized strains contained an average of 11 ± 3 spacers per allele (mean ± SD) compared to fecal strains which had 13 ± 5 spacers per allele (Fig. 2). These values were not statistically significantly different (*P *=* *0.08, Mann–Whitney Rank Sum Test). Using spacer count as a proxy for CRISPR activity (Gophna et al. 2015), it appears that naturalized and fecal strains have similar immunity to exogenous DNA. Reduced CRISPR immunity may facilitate the acquisition of environmentally adaptive genetic traits through the uptake of foreign DNA. This mechanism has been proposed to facilitate acquisition of virulence factors among pathogenic strains (Toro et al. 2014; Garcia‐Gutierrez et al. 2015). While the data from the current study suggest that CRIPSR‐mediated immunity (or lack thereof) does not play a major role in environmental adaptation among naturalized strains, this interpretation should be viewed cautiously as the number of naturalized and fecal strain used in this study was relatively small. It is therefore recommended that future studies in other watersheds should include larger numbers of strains, particularly given the tremendous diversity of CRISPR1 alleles observed among *E coli* strains.

**Figure 3:**
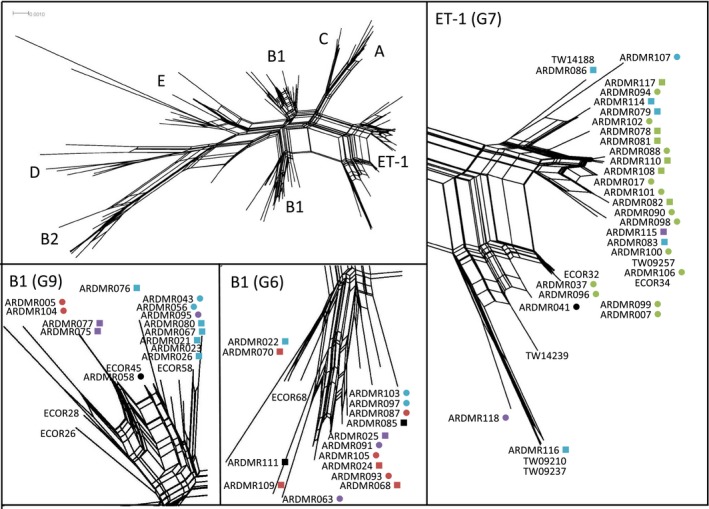
Phylogenetic relationships of naturalized and fecal *E. coli* strains based on MLST (Whittam typing scheme). The top left panel represents the Splitstree phylogenetic network of phylogroup B1 strains in relation to other major phylogroups. Major phylogenetic groups are labeled. The three panels on the right and the bottom of the figure are enlargements of the ET‐1 clade, and two distinct phylogroup B1 clades. Strain ID's are followed by a symbol representing the source of isolation (naturalized, circle; fecal, square), and the CRISPR relatedness group (G6, red; G7, green; G8, purple; G9, teal; ungrouped, black; Touchon et al. 2011). Representative phylogroup B1 ECOR strains and clade ET‐1 strains (denoted by the prefix TW) were also included.

Previous examination of *E. coli* CRISPRs indicates that certain alleles are predominantly associated with specific phylogroups (Touchon et al. 2011). Among phylogroup B1strains, four major distinct groups of alleles, with almost completely different spacer repertoires, have been previously identified (herein referred to as G6 to G9, as designated previously). The majority of CRISPR alleles from the current study belonged to one of the four groups (Fig. 1), and all four allele groups were found among naturalized strains and fecal strains. Four alleles were uncategorized. To examine CRISPR alleles in the context of phylogenetic relatedness, the phylogeny was reconstructed based on MLST. Several strains from other major phylogroups were also included in the reconstruction (Table S1). Among the phylogroup B1 strains, three distinct clades, including the previously identified naturalized ET‐1 clade (Walk et al. 2007), were observed (Fig. 3). Similar phylogenetic structure has been reported among phylogroup B1 strains from soil (Bergholz et al. 2011), although no attempt was made to see if the clades in this current study corresponded with those of the previous study. Conspicuously, the different CRISPR allele groups tended to be conserved among strains of the same phylogenetic clade. For example, all strains with G7 alleles belonged to the ET–1 clade, while seven of the nine strains with G6 alleles clustered in a second phylogroup B1 clade, and half of the strains with G9 alleles clustered in a third phylogroup B1 clade. Some strains did not group with their respective clade. This is likely due to genetic recombination, which is noted to be especially common among phylogroup B1 strains (Almendros et al. 2014).

Looking specifically at the distribution of the different CRISPR alleles, it is noteworthy that the majority of strains that have CRISPR G7 alleles (13 of 19 or 68%) were naturalized. Conversely, the majority strains with CRISPR G9 alleles were of fecal origin including 11 of 16 strains (69%). CRISPR G6 and G8 alleles were evenly distributed among naturalized and fecal strains. This indicates that while naturalized strains are genetically diverse, there appeared to be a bias toward naturalized strains having CRIPSR G7 alleles rather than G9 alleles (*P *=* *0.04, Fisher's exact test). This is consistent with notion that the ET‐1 clade (in which all CRISPR G7 alleles are found) is a naturalized clade found in aquatic environments (Walk et al. 2007). Regardless of the bias, CRISPR G7 alleles were not specific to naturalized strains, and therefore not useful as a marker for identifying naturalized strains.

## Conclusion

Despite the tremendous genetic diversity among the strains of both ecotypes, the observation that fecal and naturalized *E. coli* strains possess largely similar CRISPR spacer repertoires suggests these strains likely have a shared history of encounter with exogenous genetic elements. The most parsimonious explanation is that the strains were derived from common ancestral lineages and/or were from the same fecal sources. Likewise, MLST data also supports that naturalized phylogroup B1 strains are not genetically divergent from fecal strains, but rather, appear to represent a continuum within the global *E. coli* population. From a practical perspective, the large variation among individual strains will preclude the use of CRISPRs as typing markers for identifying naturalized populations.

## Conflict of Interest

The author declares no conflict of interest.

## Supporting information


**Table S1.** Isolation source, sequence type (ST), and MLST data for additional non‐phylogroup B1 strains analyzed in this study.

**Table S2.** Characteristics of CRISPR alleles from naturalized and fecal *E. coli* strains.
