# Supplementary material for: CRISPR1 analysis of naturalized surface water and fecal Escherichia coli suggests common origin
Source: Microbiologyopen. 2016 Mar 22;5(3):527–33. doi: 10.1002/mbo3.348 (PMC4906003; doi:10.1002/mbo3.348)
Supplement: Supplementary file 1 — Table S1. Isolation source, sequence type (ST), and MLST data for additional non‐phylogroup B1 strains analyzed in this study. [file MBO3-5-527-s001.docx]

**Table S1.** Isolation source, sequence type (ST), and MLST data for additional non-phylogroup B1 strains analysed in this study.

|  |  |  |  | MSLT allele numbers^a^ | | | | | | |
| --- | --- | --- | --- | --- | --- | --- | --- | --- | --- | --- |
| Strain ID | Source | Phylogroup | ST | *aspC* | *clpX* | *fadD* | *icdA* | *lysP* | *mdh* | *uidA* |
| ARDMR008 | Sediment | E | 1101 | 11 | 2 | 110 | 104 | 18 | 194 | 262 |
| ARDMR009 | Sediment | B2 | 28 | 28 | 33 | 31 | 36 | 4 | 36 | 25 |
| ARDMR013 | Surface water | E | 1102 | 11 | 190 | 231 | 208 | 19 | 112 | 263 |
| ARDMR015 | Sediment | B2 | 1103 | 24 | 24 | 232 | 37 | 4 | 11 | 247 |
| ARDMR018 | Cow | C | 88 | 32 | 12 | 61 | 12 | 1 | 12 | 12 |
| ARDMR019 | Cow | A | 171 | 3 | 3 | 1 | 1 | 1 | 1 | 1 |
| ARDMR027 | Cow | E | 1104 | 174 | 13 | 22 | 209 | 9 | 20 | 264 |
| ARDMR028 | Cow | B2 | 29 | 21 | 24 | 10 | 8 | 17 | 11 | 25 |
| ARDMR031 | Cow | D | 772 | 97 | 96 | 147 | 73 | 30 | 163 | 43 |
| ARDMR032 | Cow | E | 1105 | 69 | 67 | 29 | 104 | 2 | 8 | 265 |
| ARDMR033 | Surface water | A | 171 | 3 | 3 | 1 | 1 | 1 | 1 | 1 |
| ARDMR035 | Surface water | A | 171 | 3 | 3 | 1 | 1 | 1 | 1 | 1 |
| ARDMR036 | Surface water | C | 88 | 32 | 12 | 61 | 12 | 1 | 12 | 12 |
| ARDMR038 | Surface water | D | nd | 25 | 142 | 58 | 59 | 39 | 33 | 170 |
| ARDMR039 | Surface water | E | 617 | 119 | 132 | 29 | 77 | 18 | 124 | 102 |
| ARDMR040 | Surface water | B2 | 1106 | 28 | 50 | 31 | 210 | 17 | 195 | 7 |
| ARDMR045 | Surface water | D | nd | 25 | 140 | 125 | 59 | 39 | 8 | 43 |
| ARDMR046 | Surface water | C | nd | 10 | 12 | 2 | 12 | 1 | 33 | 12 |
| ARDMR048 | Surface water | E | 1107 | 5 | 5 | 233 | 38 | 1 | 195 | 1 |
| ARDMR049 | Surface water | E | nd | 11 | 2 | 22 | 136 | 19 | 2 | 182 |
| ARDMR052 | Surface water | B2 | nd | 85 | 33 | 10 | 74 | 17 | 11 | 115 |
| ARDMR053 | Surface water | B2 | nd | 71 | 28 | 102 | 74 | 40 | 33 | 72 |
| ARDMR054 | Surface water | C | 86 | 10 | 12 | 2 | 12 | 1 | 12 | 12 |
| ARDMR055 | Surface water | E | 1109 | 26 | 2 | 12 | 77 | 128 | 13 | 267 |
| ARDMR057 | Surface water | E | 1110 | 26 | 2 | 235 | 77 | 18 | 112 | 156 |
| ARDMR060 | Surface water | C | nd | 10 | 12 | 2 | 12 | 1 | 35 | 12 |
| ARDMR069 | Cow | A | 468 | 3 | 3 | 1 | 1 | 1 | 8 | 1 |
| ARDMR071 | Cow | B2 | nd | 21 | 24 | 10 | 8 | 17 | 8 | 25 |
| Abbreviations: nd, not determined; ST, sequence type. | | | | | | | | | | |
| ^b^Allele numbers according to Shigatox EcMLST database. | | | | | | | | | | |
