# Supplementary material for: CRISPR1 analysis of naturalized surface water and fecal Escherichia coli suggests common origin
Source: Microbiologyopen. 2016 Mar 22;5(3):527–33. doi: 10.1002/mbo3.348 (PMC4906003; doi:10.1002/mbo3.348)
Supplement: Supplementary file 2 — Table S2. Characteristics of CRISPR alleles from naturalized and fecal E. coli strains. [file MBO3-5-527-s002.docx]

**Table S2.** Characteristics of CRISPR alleles from naturalized and faecal *E. coli* strains.

|  | **Naturalized**  ***E. coli*** | **Faecal**  ***E. coli*** |
| --- | --- | --- |
| Number of strains | 29 | 27* |
| Total number of spacers for all strains | 317 | 341 |
| Number of distinct spacers | 109 | 120 |
| Number of strain-specific spacers | 35 | 31 |
| Number of common spacers (found in two or more strains) | 74 | 89 |
| Number of alleles | 20 | 21 |
| Number of alleles with unique spacers | 8 | 9 |

*Strain ARDMR023 was omitted from analysis as it lacked a CRISPR1 sequence
